# A Comparative Study on the Effects of Spray Coating Methods and Substrates on Polyurethane/Carbon Nanofiber Sensors

**DOI:** 10.3390/s23063245

**Published:** 2023-03-19

**Authors:** Mounika Chowdary Karlapudi, Mostafa Vahdani, Sheyda Mirjalali Bandari, Shuhua Peng, Shuying Wu

**Affiliations:** 1 School of Engineering, Macquarie University, Sydney, NSW 2109, Australia; 2 School of Mechanical and Manufacturing Engineering, University of New South Wales, Sydney, NSW 2052, Australia

**Keywords:** strain sensors, electro-spray, air-spray, electrospinning, polyurethane/carbon nanofibers

## Abstract

Thermoplastic polyurethane (TPU) has been widely used as the elastic polymer substrate to be combined with conductive nanomaterials to develop stretchable strain sensors for a variety of applications such as health monitoring, smart robotics, and e-skins. However, little research has been reported on the effects of deposition methods and the form of TPU on their sensing performance. This study intends to design and fabricate a durable, stretchable sensor based on composites of thermoplastic polyurethane and carbon nanofibers (CNFs) by systematically investigating the influences of TPU substrates (i.e., either electrospun nanofibers or solid thin film) and spray coating methods (i.e., either air-spray or electro-spray). It is found that the sensors with electro-sprayed CNFs conductive sensing layers generally show a higher sensitivity, while the influence of the substrate is not significant and there is no clear and consistent trend. The sensor composed of a TPU solid thin film with electro-sprayed CNFs exhibits an optimal performance with a high sensitivity (gauge factor ~28.2) in a strain range of 0–80%, a high stretchability of up to 184%, and excellent durability. The potential application of these sensors in detecting body motions has been demonstrated, including finger and wrist-joint movements, by using a wooden hand.

## 1. Introduction

Flexible and stretchable sensors are attracting tremendous attention due to their enormous potential in a variety of applications such as motion detection, health monitoring, and human-machine interfaces [1,2]. Conventional sensors made of metals and semi-conductors are rigid, brittle, and strenuous to wear, making them challenging for use as wearable sensors [3]. On the other hand, elastic polymer nanocomposite-based sensors are lightweight, soft, and can be made hypoallergenic [4,5,6]. Due to their high flexibility, they can be fixed on non-flat surfaces (any part of our body) and/or attached to clothing for long-term health monitoring. Sensors based on different working mechanisms have been developed including piezocapacitive [7], piezoresistive [8], piezoelectric [9], and triboelectric [10]. Among these, piezoresistive sensors have attracted considerable interest due to the relatively simple read-out systems and advantages such as high sensitivity and high stretchability [2,11].

Flexible piezoresistive sensors are usually made of an elastic substrate or matrix in combination with conductive fillers [8,9,10,11,12,13,14,15,16]. Conductive materials such as carbon black [12], metal nitrides/carbides (MXenes) [13], carbon nanotubes [14], carbon nanofibers (CNFs) [15,16], silver nanowires (AgNWs) [17] and gold nanowires [18], and graphene [19] are most commonly used. In particular, the one-dimensional conductive carbon nanomaterials (e.g., carbon nanotubes or nanofibers) were extensively studied due to their high electrical conductivity and mechanical properties, as well as high stability [20]. Moreover, characteristics such as low cost and ease of fabrication must also be considered while developing wearable electronics [21]. CNFs are one-dimensional carbon nanomaterials that have high thermal and electrical conductivity [22] and possess various advantages such as low production cost and ease of functionalization, compared to other carbon nanomaterials (CNTs and graphene) [23]. Many recent studies demonstrated the potential use of this material in preparing high-performance wearable electronics [24,25,26,27,28].

To achieve high flexibility and stretchability, elastic polymers such as Ecoflex, natural rubber, and thermoplastic polyurethane (TPU) are commonly utilized [29]. Among them, TPU can be readily made into different forms such as electrospun nanofibers and solid thin films, thus offering tremendous possibility in terms of creating high-performance sensors. For instance, Lu et al. developed a strain sensor in a sandwich structure using AgNWs and electrospun TPU mats via a vacuum filtration process. The sensor exhibited high durability with a sensitivity (gauge factor, GF) of ~12 and a workable strain range of up to around 80% [30]. Zhang et al. produced wearable sensors based on MXenes and TPU, encapsulated by polydimethylsiloxane (PDMS). The TPU substrate was obtained using the solution casting method and the thickness of the film was controlled to 1 mm. MXenes were transferred to the substrate by ultrasonic treatment for 30 min to ensure uniform dispersion. PDMS was then used to encapsulate the sensor. This sensor was capable of detecting a very low strain range of less than 0.005%, has a high durability over 3000 cycles, a fast response (response time ~120 ms), and a sensing range of up to 90% [31].

Different techniques were developed to deposit the conductive sensing materials on the elastic substrate, including electro-spray and air-spray. Air-spray is a process of transferring coating materials (solution and/or particle dispersion) to a surface by pressurized air. In this process, the liquid is forced through the nozzle and forms small and fine droplets by using gas. It is an economical, time efficient, and easy-to-use process and a promising method for making films with a large conductive area [32]. Electro-spray, similar to electro-spinning, is an electro-hydrodynamic process in which an electrostatic force is used to spray a suspension or solution. Process parameters play significant roles in determining the properties of the resultant specimens. Therefore, these parameters must be determined [33].

Towards the goal of developing highly sensitive, stretchable, and durable strain sensors, this work investigates the influences of the elastic substrate (i.e., electrospun or solid thin film TPU) and deposition methods (i.e., air-spray and electro-spray) on the sensing performance of the resultant TPU/CNFs sensors. Porous TPU nanofiber mats were prepared by electrospinning while solid TPU thin films with similar thicknesses were solution cast. CNFs were then either air-sprayed or electro-sprayed onto the TPU substrates. The sensitivity and stretchability of the resultant TPU/CNF sensors were characterized and compared. Moreover, the effects of the CNFs’ area density on the sensing performance (sensitivity and stretchability) were examined. The sensing mechanism was studied by observing the structural changes when subjected to cyclic tensile loading–unloading, which was also correlated with their sensing performance. Lastly, the potential application of the sensors in motion detection was demonstrated on a wooden hand, including wrist and finger bending movements.

## 2. Experimental Section

### 2.1. Materials

Carbon nanofibers (CNFs) were purchased from Applied Sciences Inc. (grade: PR-24-XT-HHT and Pyrograf-III). Isopropyl alcohol (IPA), tetrahydrofuran (THF), and dimethylformamide (DMF) were obtained from Chem-supply Australia and polyvinylpyrrolidone (PVP) was bought from Sigma-Aldrich Australia. The RE-FLEX TPU pellets used for fabricating the nanofiber TPU substrate was obtained from Townsend Chemicals Proprietary LTD.

### 2.2. Fabrication of TPU/CNFs Strain Sensors

**Figure 1:**
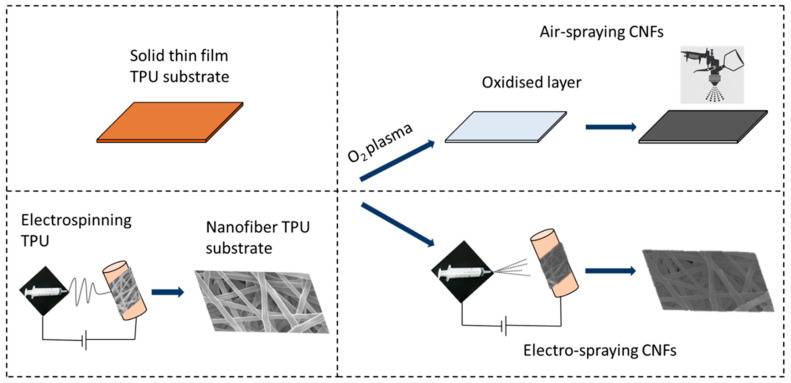
Schematic of fabricating sensors based on S-TPU and NF-TPU substrates using either the air-spray or electro-spray technique.

Four different sets of sensors were prepared, including sensors based on solid TPU thin film substrate with CNFs being air-sprayed or electro-sprayed onto the substrate and sensors based on electrospun TPU with CNFs being air-sprayed or electro-sprayed onto the substrate (Table 1). The abbreviations of these four sets of sensors are listed in Table 1. The detailed preparation procedures of the sensors are given below and illustrated in Figure 1.

*TPU substrate preparation*: The TPU nanofiber mats were obtained using an electrospinning process. Firstly, the TPU pellets were dissolved in DMF and THF mixed in a volume ratio of 1:3. The dissolution was completed by magnetic stirring at 300 rpm and 60 °C for 4 h. A TPU solution of three TPU concentrations was prepared and compared, including 6 wt.%, 8 wt.%, and 10 wt.%. The electrospinning process was conducted on a NanoSpinner electrospinning device (Inovenso NS+ plus, 220 V, 50/60 Hz). A syringe with a 10 mL capacity was used to load the TPU solution. The influences of the different spinning parameters were examined, including the TPU solution concentration, voltage, flow rate, and spinneret-to-collector distance. The low voltage cannot produce nanofibers due to low electric force while a voltage beyond the critical value results in an unstable jet [34]. After trying voltages of 20 kV, 23 kV, and 25 kV, it was noted that the 23 kV voltage enabled the effective production of uniform nanofibers. The influence of the flow rate, TPU concentration, and spinneret-to-collector distance will be discussed in detail in Section 3.

*Spray coating for depositing CNFs on TPU*: CNFs are deposited on the substrates (solid TPU thin film or electrospun TPU nanofibers) using two different deposition processes: (a) air-spray and (b) electro-spray. To perform the coating, CNFs were first dispersed in IPA by using PVP as the dispersion agent. The CNF concentration is 0.1 wt.% while the PVP of the same concentration (0.1 wt.%) was used. This mixture was sonicated for about 1 h using an ultrasonic probe-sonicator. Sensors with CNFs of different area densities can be produced by varying the volume of the CNF solution sprayed on the substrate of the same area.

In the air-spray deposition process, the substrate was surface treated by using O_2_ plasma for about 1 min using a plasma surface treatment machine (Zepto, Diener Electronics, Ebhausen, Germany) to promote the adhesion with the CNFs. The air-spray technique was performed using a Blackridge airbrush kit which has a nozzle diameter of 0.3 mm and the operating pressure was 18–20 psi at a distance of 15 cm. After air-spray, the specimen was heat treated at 60 °C for about 1 h and thin copper wires were attached on either side using silver paste which acts as leads for measuring the resistance during piezoresistivity tests.

The electro-spray was carried out by using a NanoSpinner electrospinning device (Inovenso NS+ plus, 220 V, 50/60 Hz, Inovenso, Cambridge, MA, USA), the same device used to obtain the TPU nanofiber substrate. The CNF solution is loaded into the syringe with a 10 mL capacity. The drum collector to the tip of the spinneret distance, the voltage, drum collector rotating speed, and flow rate were determined to be 10 cm, 23 kV, 300 rpm, and 10 mL/h, respectively, to ensure uniform coating and no liquid dripping. It should be mentioned that both TPU substrates were not surface treated for this electro-spray method because no big droplets were observed during the spraying process and a very uniform coating can be achieved by adjusting the flow rate of electro-spray. After spraying, the samples were peeled off from the drum collector and heat treated at 60 °C for about 1 h. Thin copper wires were then attached as electrodes for a resistance measurement.

### 2.3. Characterisation

#### 2.3.1. Morphology

The morphology of the solid thin film and TPU nanofiber substrates were studied using Field Emission Scanning Electron Microscopy (FESEM, Nova NanoSEM, FEI company, Hillsboro, OR, USA). Platinum coating was conducted before the examination. To understand the structural changes of the TPU/CNFs sensors, the samples were stretched to different levels of strains (up to 100%) and the microstructure of the CNFs sensing layer was examined using SEM and an optical microscope.

#### 2.3.2. Piezoresistivity Measurements

The piezoresistivity of the TPU/CNFs was characterized by measuring the resistance changes when subjected to tensile stretch. By using a custom-made linear stage device, the samples were imposed by cyclic and quasi-static tension. To measure the sensor responses to cyclic loading–unloading, 8 cycles were applied with peak-strain ranging between 1% to 650% (depending on the sample failure strain) at a velocity of 1 mm/s. Three samples were tested to investigate the repeatability of the sample performance. The sensitivity of the sensors was measured by applying linear regression to the data obtained from the tensile test and by analyzing the changes in the resistance and strain (ε). The durability was measured by examining the sensors under 3000 cyclic tensile loading and unloading cycles at a velocity of 1 mm/s (equivalent to strain rate of 20%/s). The sensor’s piezoresistivity under different stretching velocities from 1 mm/s, 5 mm/s, 9 mm/s, to 13 mm/s was also studied while the response time was examined by stretching the sample to 50% strain at a velocity of 13 mm/s (the highest velocity the equipment can accurately reach).

## 3. Results and Discussion

### 3.1. Effects of Electrospinning Parameters on Nanofiber Production

**Figure 2:**
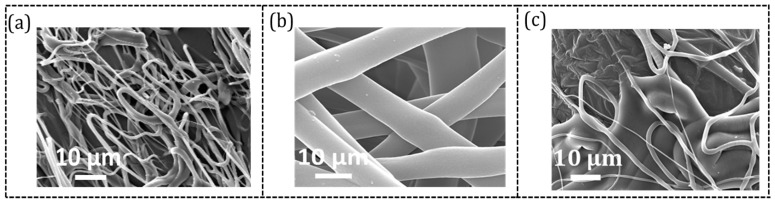
SEM images of TPU nanofibers electrospun from TPU solution of different concentrations: (**a**) 6 wt.%, (**b**) 8 wt.%, and (**c**) 10 wt.%.

The concentration of the TPU solution plays a crucial role in producing uniform nanofibers. Figure 2 shows the SEM images of electrospun nanofibers produced from a TPU solution of three different concentrations, namely, 6 wt.%, 8 wt.%, and 10 wt.%. The flow rate and the spinneret-to-collector distance for the electrospinning process were 2.5 mL/h and 10 cm, respectively. Figure 2b indicates nanofibers made from an 8 wt.% TPU solution displaying a uniform diameter without forming any beads. However, when 10 wt.% TPU solution was used, nanofibers of non-uniform diameters were obtained, as shown in Figure 2c. Notably, there were droplets forming at the nozzle tip which were not stretched (no Taylor cone was observed) and spun into fibers. The same phenomenon occurred for the 6 wt.% concentration and non-uniform fibers were generated (Figure 2a). Hence, a TPU solution with an 8 wt.% concentration was used to fabricate the NF-TPU substrate.

**Figure 3:**
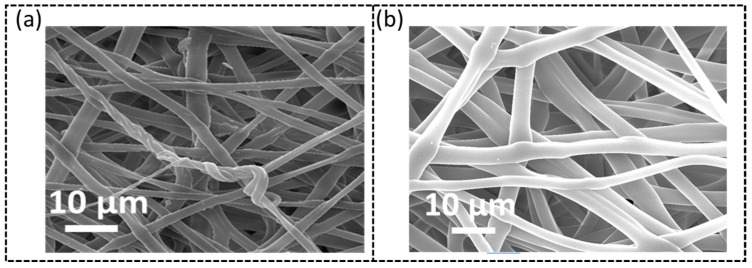
SEM images of TPU nanofibers electrospun from 8 wt.% TPU solution at different distances between the spinneret and drum collector: (**a**) 15 cm and (**b**) 10 cm.

Another parameter that plays a significant role in the production of uniform nanofibers and needs to be determined is the spinneret-to-collector distance. Three different distances were studied (5 cm, 10 cm, and 15 cm) while the flow rate and the TPU solution concentration were 2.5 mL/h and 8 wt.%, respectively. The structural morphology of the resultant nanofibers was analyzed by using SEM. It was also noticed that a 5 cm distance was too small for the liquid to stretch into nanofibers. Moreover, from Figure 3, we can see that although there is no noticeable difference between the two in terms of uniformity, the sample electrospun at a larger distance (15 cm, Figure 3a) has a smaller diameter (average diameter ~0.34 ± 0.1 μm) as compared to those obtained at a shorter distance (10 cm, Figure 3b, average diameter ~0.47 ± 0.07 μm). Moreover, it was found that thinner diameter nanofibers can lead to early mechanical failure. Therefore, the spinneret-to-collector distance was determined to be 10 cm.

The third parameter studied is the flow rate of liquid feeding during electrospinning. A low flow rate results in the instability of the jet while a high flow rate leads to bead formation due to less volatility of the solution causing the bonding between the fibers [35]. Three flow rates, 1, 2.5, and 4 mL/h, were studied in this work. It was found that the jet was unstable, and droplets were formed when 1 and 4 mL/h were used. From Figure 2b and Figure 3b, we can see that the flow rate of 2.5 mL/h did not cause any bead formation and that the TPU nanofibers are uniform. Thus, for this study, a flow rate of 2.5 mL/h, TPU concentration of 8 wt.%, and a spinneret-to-drum collector distance of 10 cm was chosen for electrospinning TPU.

### 3.2. Piezoresistivity of the TPU/CNFs Sensors

**Figure 4:**
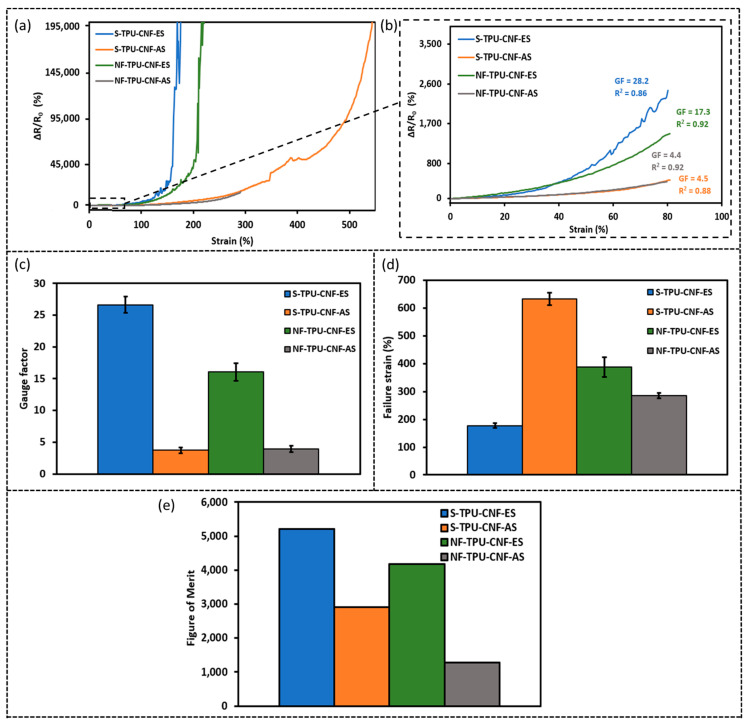
(**a**) Relative resistance change (ΔR/R_0_ (%)) versus strain (%) of 0.65 mg/cm^2^ area density strain sensors up to failure strain, (**b**) ΔR/R_0_ (%) of sensors shown in (**a**) up to 80% strain; (**c**) sensitivity, (**d**) stretchability; and (**e**) figure of merit for the four types of sensors.

Sensors fabricated using different spray methods and different substrates were prepared and characterized. It should be mentioned that the area density of CNFs deposited on the TPU substrate discussed in this Section is 0.65 mg/cm^2^. Figure 4a,b shows the relative resistance change (%) versus the tensile strain (%) of these sensors. The resistance increases upon increasing the tensile strain up to its failure strain and the resistance increase rate also increases at larger strains. The non-linear relationship in the curves of relative resistance change (ΔR/R_0_) versus the strain is often observed (particularly in a large strain range) in piezoresistive sensors based on elastic polymer nanocomposites [36]. The reason for this non-linearity in the large strain may be due to the tunneling resistance which increases exponentially upon increasing the distance between two conductive particles [37], as well as stress relaxation and creep [38]. The gauge factor (an indicator of the sensitivity) is defined as the slope in ΔR/R_0_ versus the strain curve in the strain range <80%. It should be also noted that the stretchability is the failure strain (electrical failure where the resistance is too high to be measured by the used multimeter with 1 GΩ capacity). Figure 4c,d summarizes the sensitivity and stretchability of these four types of sensors while Figure 4e gives the corresponding figure of merit (= GF × Stretchability). It can be seen that S-TPU-CNF-ES shows the highest sensitivity with a gauge factor of ~28.2 but the lowest stretchability of ~184% strain. In contrast, S-TPU-CNF-AS has a high stretchability of up to 660% but the sensitivity is the lowest with a gauge factor of ~4.4. Moreover, it is noticeable that the samples with CNFs electro-sprayed generally showed higher sensitivity compared to air-sprayed samples. However, the influence of substrates, whether solid TPU or electrospun TPU nanofibers, on sensitivity and stretchability is not explicit.

The sensors developed in this research are intended to be used for human motion detection. Therefore, the sensors must have a high stretchability (>~50%) to be capable of detecting human motions as the strain associated with some human motions (e.g., joint bending) can reach about 50% [11]. Thus, the sensor made from solid TPU thin film with CNFs electro-sprayed, which shows the highest sensitivity and sufficient stretchability, will be the focus in the following studies.

### 3.3. Influences of Electro-Spray Parameters and CNF Area Density

There are several parameters that need to be determined during electro-spray, including the applied voltage, spinneret-to-collector distance, and flow rate, as well as the concentration of the CNFs IPA dispersion. The CNF concentration was set to be 1 wt.% since a stable dispersion (no clear agglomerates were observed after 12 h) can be achieved at this concentration. The electro-spray voltage and spinneret-to-collector distance were determined by observing the sprayed CNFs to ensure that there are no obvious droplets (agglomerates) during spraying. Three voltages, i.e., 20 kV, 23 kV, and 25 kV, and three spinneret-to-collector distances, i.e., 5 cm, 10 cm, and 15 cm, were examined. It was noted that a 23 kV voltage and 10 cm spinneret-to-collector distance could produce uniform electro-spray without any droplet formation on the substrate. However, the influence of the flow rate on electro-sprayed CNFs on the substrates cannot be readily seen based on the appearance of the specimens.

**Figure 5:**
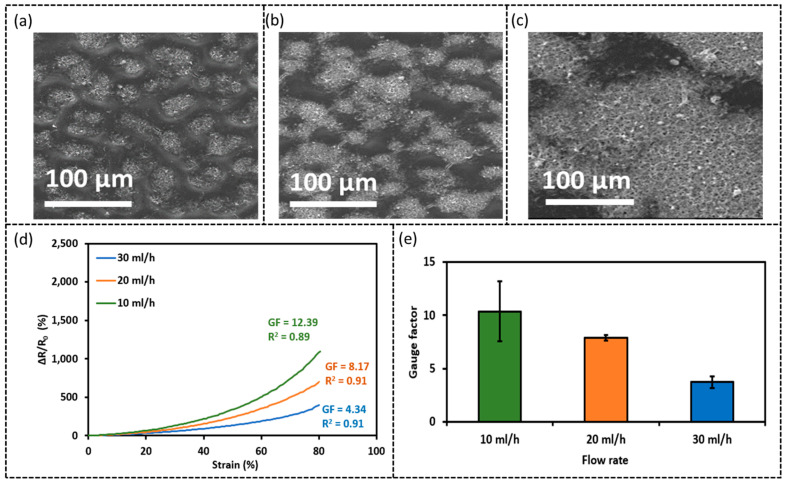
SEM images of sensors prepared with different flow rates for electro-spray (**a**) 10 mL/h, (**b**) 20 mL/h, (**c**) 30 mL/h, (**d**) relative resistance change (%) vs. tensile strain (%), and (**e**) gauge factor derived from (**d**).

Thus, the piezoresistivity of specimens prepared by three different flow rates, i.e., 10, 20, and 30 mL/h, were compared. It should be noted that the area density of CNFs was fixed at 0.35 mg/cm^2^ in all these three cases. From Figure 5a–c, we can see that the CNFs layer sprayed at TPU thin film has a bright area (CNFs islands) separated by a dark area where CNFs of a much lower density are observed. Upon increasing the flow rate, the area with sparse CNFs seems to be decreased, indicating that CNFs are distributed more uniformly across the entire area. Figure 5d shows the relative resistance change (ΔR/R_0_) under tensile strain up to 80% strain. It is seen that the sensor prepared using a lower flow rate shows a higher gauge factor (Figure 5e), which is calculated using linear regression fit, indicating higher sensitivity. The larger resistance changes when subjected to the same level of strain may be attributed to the difference in the CNF distribution. When sprayed at the low flow rate, the CNF-rich islands are bridged by the CNF-sparse areas which form a continuous network. It is believed that this continuous network with a low centration of CNFs plays a dominant role in contributing to the piezoresistivity. It has been demonstrated that a lower concentration of conductive nanomaterials usually results in higher sensitivity because it is easier to destruct the conductive networks if the nanomaterials are initially less overlapped and have a lower contact area [11,37,39]. Hence, a 10 mL/h flow rate was selected for electro-spray due to the higher sensitivity.

**Figure 6:**
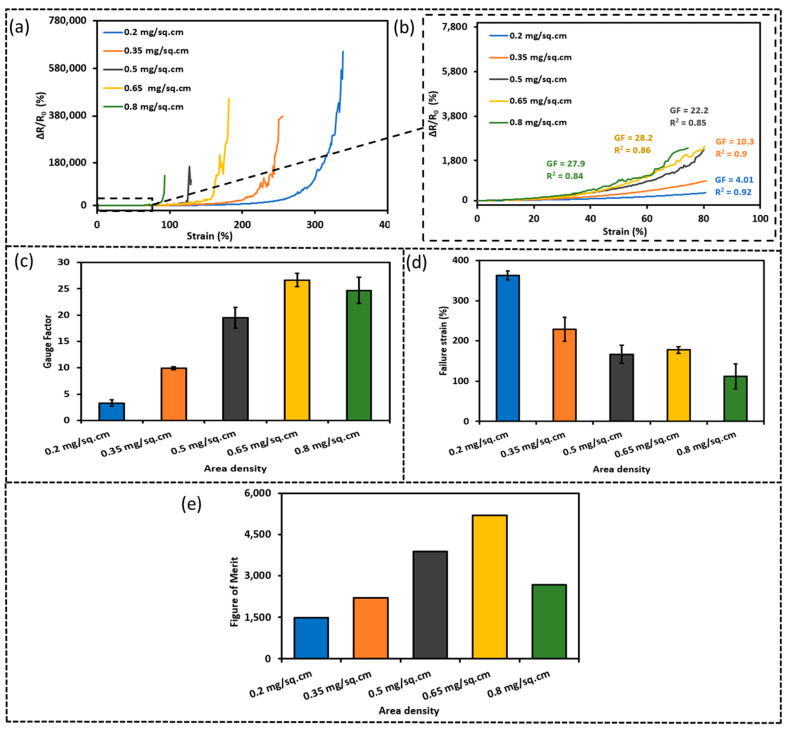
Relative resistance change (ΔR/R_0_ (%)) versus strain (%) for sensors made of CNFs of different area densities: (**a**) up to failure strain, (**b**) in the low strain range (0%–80%), (**c**) sensitivity, (**d**) stretchability of S-TPU-CNF-ES sensors, and (**e**) figure of merit of the corresponding sensor with different area density.

Moreover, the area density of CNFs is believed to be critical and the piezoresistivity of sensors having CNFs of different area densities was investigated. Notably, the area density was evaluated based on the quantity of CNF solution spray-coated over the TPU substrate of a certain area. Figure 6a shows the relative change in resistance versus the tensile strain for S-TPU-CNF-ES sensors of area densities 0.2, 0.35, 0.5, 0.65, and 0.8 mg/cm^2^ up to the failure strain while Figure 6b gives the relative resistance changes in the strain range 0–80%, based on which the gauge factor and linearity (R^2^) values are obtained. Figure 6c,d shows the gauge factors and stretchability of these sensors and Figure 6e gives the corresponding figure of merit. Notably, the sensor made of 0.65 mg/cm^2^ CNFs has the highest sensitivity (GF ~ 28.2). The gauge factor increased gradually from GF ~ 4.1 at 0.2 mg/cm^2^ to GF ~ 28.2 at 0.65 mg/cm^2^ and then decreased to GF ~ 28 at 0.8 mg/cm^2^. In addition, upon increasing the area density (thickness of the deposition), the cracks tend to form more easily at lower strains, which may account for the increase in GF. In contrast, the stretchability decreased from 361% at 0.2 mg/cm^2^ down to 112% at 0.8 mg/cm^2^. By considering the sensitivity and stretchability, the sensor with CNFs of 0.65 mg/cm^2^ having 184% stretchability and 28.2 GF was chosen for the studies below.

### 3.4. Durability, Response Time, and Responses to Different Strain Rates

**Figure 7:**
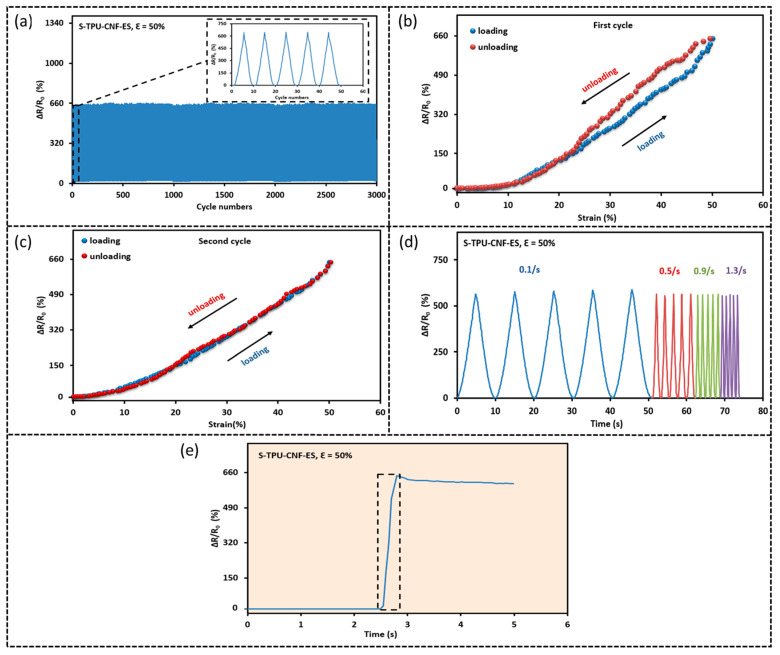
Relative resistance change (ΔR/R_0_ (%)) versus strain (%) for S-TPU-CNF-ES: (**a**) 3000 cycles of stretching–releasing with a peak strain of 50%; (**b**,**c**) hysteresis curves for the first (**b**) and the second (**c**) cycle with a peak strain of 50%; (**d**) the effect of strain rate on the resistance changes for 5 cyclic stretching–releasing with a peak strain of 50%; (**e**) the response time of the sensor by applying 50% strain at a strain rate of 130%/s.

To study the durability, 3000 loading–unloading cycles of up to 50% were applied to the S-TPU-CNF-ES sensor (0.65 mg/cm^2^). Figure 7a presents the relative resistance changes of S-TPU-CNF-ES. The resistance increases by 655% under 50% strain. Moreover, minimal variations (variation in the maximum ΔR/R_0_ at the peak strain for the first and the last cycle = 1.98%) in the relative resistance change even after 3000 cycles of stretching–releasing are detected, indicating that the samples show excellent durability. The ΔR/R_0_ (%) versus strain curves for the first and second cycles are presented in Figure 7b,c. The hysteresis was estimated to be around 7.2% based on a previously reported method [36] and it was slightly higher than a previously reported hydrogel-based sensor [40]. Figure 7d shows that the strain rate has negligible effects on the sensor as it exhibits consistent resistance changes when subjected to the tensile strain applied at different rates. The response time is another key parameter that indicates the response rate of the sensor when subjected to an externally applied strain. A shorter response time means the sensor is more responsive. Based on Figure 7e which depicts the response time when a 50% strain was applied at 13 mm/s, the response time was determined to be ~30 ms, which is quicker or comparable to other sensors [41,42,43,44].

### 3.5. Piezoresistivity Mechanism

**Figure 8:**
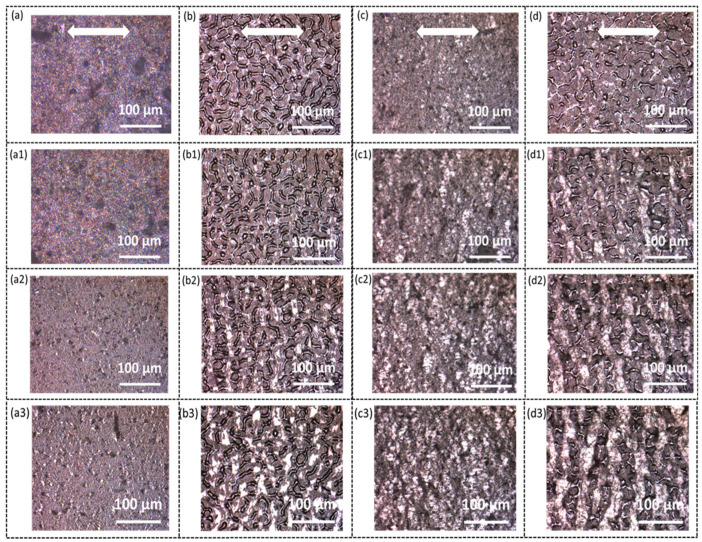
Optical microscope images of S-TPU-CNF-AS when subjected to (**a**) 0%, (**a1**) 30%, (**a2**) 60%, and (**a3**) 100% strain; S-TPU-CNF-ES when subjected to (**b**) 0%, (**b1**) 30%, (**b2**) 60%, and (**b3**) 100% strain; NF-TPU-CNF-AS when subjected to (**c**) 0%, (**c1**) 30%, (**c2**) 60%, and (**c3**) 100% strain; NF-TPU-CNF-ES when subjected to (**d**) 0%, (**d1**) 30%, (**d2**) 60%, and (**d3**) 100% strain. Notably, the stretching direction is indicated by the white arrow.

**Figure 9:**
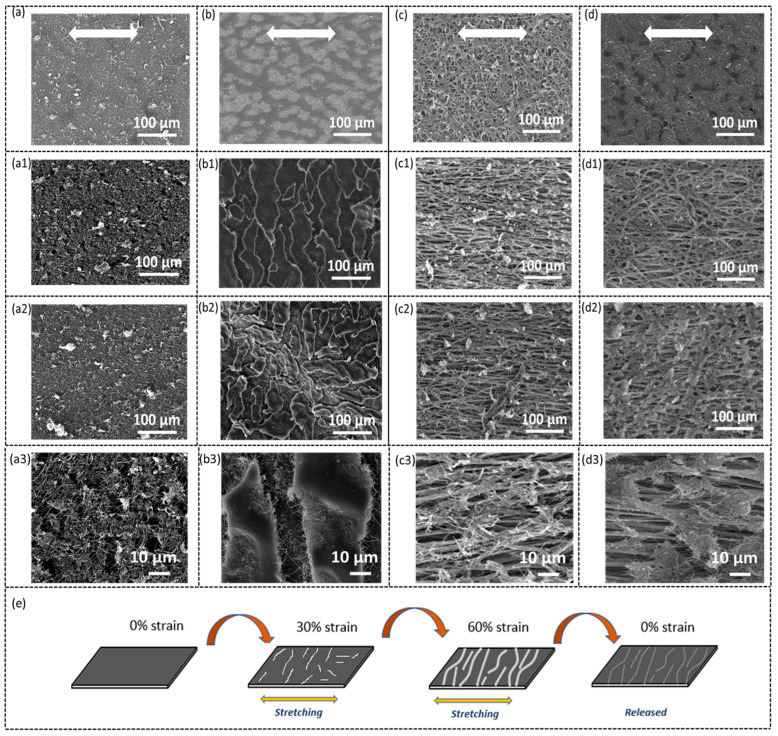
SEM images of S-TPU-CNF-AS when subjected to (**a**)0%, (**a1**) 30%, (**a2**) 60%; S-TPU-CNF-ES when subjected to (**b**) 0%, (**b1**) 30%, and (**b2**) 60% strain; NF-TPU-CNF-AS when subjected to (**c**) 0%, (**c1**) 30%, and (**c2**) 60% strain; NF-TPU-CNF-ES when subjected to (**d**) 0%, (**d1**) 30%, and (**d2**) 60% strain. (**a3**–**d3**) are the corresponding high-magnification SEM images. (**e**) Schematic of the cracking mechanism of TPU/CNFs-based sensors.

To understand the sensing mechanism and different performance between the four types of sensors, an optical microscope and SEM were used to observe the structure change of the carbon nanofibers sensing layer under stretching. Figure 8 and Figure 9 show the corresponding optical and SEM images, respectively. The stretching direction is horizontal. By comparing Figure 8a–d, it is seen that the sensors with CNFs air-sprayed and electro-sprayed show a slightly different morphology. A “Bicontinuous” structure is observed in sensors (Figure 8b,d) with CNFs electro-sprayed while those with CNFs air-sprayed display a uniform, relatively feature-less structure (Figure 8a,c). The corresponding SEM shows a similar morphology, i.e., CNFs are uniformly sprayed on the substrate when they are air-sprayed while sensors with CNFs electro-sprayed show a “bicontinuous” structure with CNFs in both dense and sparse regions (i.e., the grey and dark areas, respectively, in Figure 9b,d). This unique morphology may be due to the different drying processes during the spraying. It was noted that the air-spray technique is a much quicker process, the electro-spray and liquid droplets are much smaller, and that the solvent dries much more quickly, which may lead to much more uniform deposition on the substrate [45]. It should be also noted that the substrates (both solid TPU film and electrospun TPU nanofibers) were not treated by plasma before the electro-spraying process, which may also account for the different deposition.

When the sensors are stretched, cracks are formed in the CNFs sensing layer, as indicated by the optical micrographs (Figure 8). Cracks are especially obvious in Figure 8(b1–b3,c1–c3,d1–d3) taken from the sensor S-TPU-CNF-ES, NF-TPU-CNF-AS, and NF-TPU-CNF-ES. The cracks continue to open when the strain increases, which explains the increase in resistance when the sensors are stretched to higher strains. The formation of cracks can also be confirmed by the SEM images (Figure 9(b1–b3,c1–c3,d1–d3)). A schematic illustrating the cracking mechanism in the as-developed TPU/CNFs is shown in Figure 9e. The cracking mechanism has been recognized as a very effective sensing principle for achieving highly sensitive sensors based on conductive polymer thin films [24,25,26,46,47,48]. As for the S-TPU-CNFS-AS, cracks can be observed in Figure 9(a1–a3) at a higher magnification, although no clear cracks are seen in the optical micrographs (Figure 8(a1–a3)). Moreover, smaller crack opening (means smaller increase in resistance) is seen for S-TPU-CNFS-AS as compared to the other three types of sensors, which accounts for the sensor’s higher stretchability and lower sensitivity.

### 3.6. Application Demonstration

**Figure 10:**
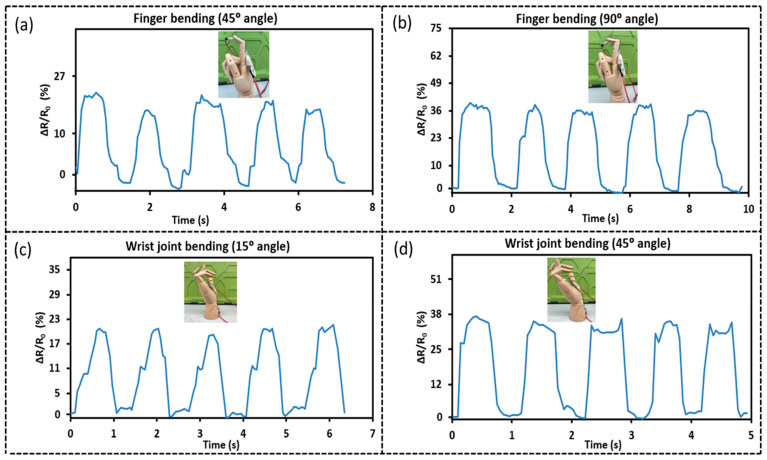
Application demonstration of the S-TPU-CNF-ES based sensor. (**a**) Finger bending at 45° angle. (**b**) Finger bending at a 90° angle. (**c**) Wrist joint bending at a 15° angle. (**d**) Wrist joint bending at a 45° angle.

To illustrate the prospective applications of the as-developed thin film sensors as wearable sensors, S-TPU-CNF-ES, with an area density of 0.65 mg/cm^2^ and showing the highest sensitivity, is examined for its ability to detect human motions to measure the finger and wrist joint bending by using a wooden hand. Detecting joint bending requires a sensor with high sensitivity as well as stretchability. This sensor succeeded in detecting these movements. The sensor was first positioned on the index finger which was then bent at a 45° and a 90° angle, respectively, as displayed in Figure 10a,b. The change in relative resistance was measured to be ~ 25% at a bending angle of 45° and 40% at a 90° bending angle, indicating a strain of 17.2% and 26.3%, respectively. These results are consistent with those reported in the literature [24]. Resistance changes associated with wrist bending at 15° and 45° angles are displayed in Figure 10c,d. The relative resistance change is 22% at 15° angle bending and 38% at 90° angle bending, indicating a strain of 9.58% and 13.7%, respectively, in good agreement with the previous report [49].

## 4. Conclusions

In conclusion, piezoresistive strain sensors with high sensitivities and broad working strain ranges were developed by the systematic investigation and comparison of the performance of sensors made of different thermoplastic polyurethane substrates (either solid thin film or electrospun nanofibers) with either electro-sprayed or air-sprayed conductive sensing layer. It is found that sensors made of electro-sprayed CNFs generally showed higher sensitivity as compared to those with air-sprayed CNFs. Meanwhile, the influence of the substrate, whether a solid or porous electrospun nanofiber, seems to be minimal as there are no obvious trends observed. The sensors made from a solid thin film TPU substrate with CNFs electro-sprayed (S-TPU-CNF-ES) at an area density of 0.65 mg/cm^2^ have demonstrated the highest sensitivity (GF ~ 28.2), a wide failure strain range (184%), high linearity (R^2^ = 86%), excellent durability over 3000 cycles, and a fast response (response time ~30 ms). This sensor successfully demonstrated its application in human movement measurement, indicating its potential use in detecting joint bending, thereby facilitating the application of these sensors in health monitoring, e-skin, human-machine interference, and soft robotics. This research provides a futuristic strategy to develop a simple yet robust technique to produce piezoresistive strain sensors that are highly sensitive and stretchable and can be used for monitoring human motions.

## Figures and Tables

**Table 1:** Sensors investigated in this study and their abbreviation.

Abbreviation	Description
S-TPU-CNF-AS	Solid TPU thin film with air-sprayed CNFs
S-TPU-CNF-ES	Solid TPU thin film with electro-sprayed CNFs
NF-TPU-CNF-AS	Nanofiber TPU with air-sprayed CNFs
NF-TPU-CNF-ES	Nanofiber TPU with electro-sprayed CNFs

## Data Availability

Not applicable.
